# Video games and board games: Effects of playing practice on cognition

**DOI:** 10.1371/journal.pone.0283654

**Published:** 2023-03-27

**Authors:** Léa Martinez, Manuel Gimenes, Eric Lambert

**Affiliations:** Centre de Recherches sur la Cognition et l’Apprentissage, Université de Poitiers, Poitiers, France; University of Milan, ITALY

## Abstract

The worldwide popularity of playing practices has led to a growing research interest in games’ impact on behavior and cognition. Many studies have already reported the benefits of both video games and board games for cognitive functions. However, these studies have mainly defined the term players according to a minimum play time or in connection to a specific game genre. No study has confronted the cognitive implications of video games and board games in the same statistical model. Thus, it remains unclear whether the cognitive benefits of play are due to play time or game type. To address this issue, in this study, we conducted an online experiment in which 496 participants completed six cognitive tests and a playing practice questionnaire. We examined the between the participants’ overall video game and board game play times and cognitive abilities. The results demonstrated significant relations between overall play time and all cognitive functions. Importantly, video games significantly predicted mental flexibility, planning, visual working memory, visuospatial processing, fluid intelligence, and verbal working memory performance, while board games were not found to predict any cognitive performance. These findings suggest that video games affect cognitive functions in specific ways compared to board games. We encourage further investigation to consider players’ individual differences through their play time and the specific features of the games they play.

## Introduction

Boasting global markets of over US$150 billion for video games [1] and over US$7 billion for board games [2], the game industry represents a major entertainment market. Indeed, the video and board game industries offer a wide range of games that are usable in various domains. The growing popularity of games markets has led to an increasing amount of research on how game practice affects human behavior and cognitive functions. A common method of carrying out such research is to compare players’ and nonplayers’ abilities. Therefore, recent studies have mainly shown that players demonstrate better cognitive performance than non-players do [3, 4]. Until now, players have generally been defined according to an overall minimum play time or to a minimum game genre-specific play time. However, player profiles are numerous and represent a wide diversity of playing practices. Moreover, a primary limitation of such research involves the lack of consideration of variability in the players’ play time and the type of game they play. Therefore, it remains unclear whether the cognitive benefits of play are due to overall play time or to specific game types.

### Play time–based definition of players

Video games’ benefits for cognitive functions have already been widely demonstrated, with a main effect on executive functions [3]. Many studies have shown that video gamers outperform non-gamers in terms of attention, visuospatial, working memory, and mental flexibility performances [5–7]. For example, one study considered visual working memory skills and found that participants who played video games for more than 5 hours per week outperformed participants who played video games for less than 5 hours per week in connection to these skills [8]. Similarly, video game training has been shown to improve attention, visuospatial, and working memory skills [9–11]. For example, playing Call of Duty for 28 hours significantly improved visual working memory performance [9]. Therefore, recent literature widely supports the beneficial effect of video gaming on cognitive and executive abilities. However, these findings are mainly based on the opposition between gamers and non-gamers as a way to compare the cognitive performance of the two groups. In this way, a first limitation to fully understanding the relationship between play and cognitive functions is the failure to consider individual differences within the gamer group [12], such as variability in gamers’ overall play time.

Most studies have focused on video gamers and defined them according to their amount of play time in the previous months (e.g., Wong & Chang, (2018) [13] defined participants who played video games for more than 4 hours a week over the past 6 months as video gamers). However, a consensus has not been reached on the amount of play time defining a video gamer. In their literature review on video games and cognitive enhancement, Choi et al. (2020) [3] listed 10 studies in which video gamers were defined based on the number of hours spent playing video games per week. In the various studies, participants were considered video gamers if they played for more than 2 hours to 15 hours per week, whereas non-gamers were defined as playing less than 1 hour to 8 hours per week. Therefore, the current way of defining gamers into two groups (i.e., gamers and non-gamers) without considering the variability of their gaming experience represents a bias. Only a few studies have focused on the effect of video gaming on cognitive functions according to specific gamers’ expertise and their play time. These studies mostly examined electronic sports (e-sports) gamers’ cognitive abilities. The term e-sports refers to the individual and collective practice of engaging in video game competitions [14]. E-sports gamers are mostly considered expert gamers, and they are sometimes professional gamers. Since e-sports gamers report higher play times than casual gamers do [5], focusing on these gamers allows the effects of intensive video game practice on cognitive functions to be studied. Professional e-sports gamers usually outperform nonprofessional, casual gamers on cognitive tests, such as visuospatial processing, visuospatial memory, and attention tests [5, 15]. For example, professional e-sports gamers who play more than 20 hours a week have shown better visuospatial processing, visuospatial memory, and attention performance compared with casual video gamers who play more than 5 hours per week [5]. The same results have been found when comparing professional and nonprofessional e-sports gamers. Professional e-sports gamers who play about 35 hours a week have been found to outperform nonprofessional e-sports gamers who play about 20 hours a week on a simple visual reaction test [15]. Thus, whether professional or not, it seems that the more time gamers spend playing video games, the higher their cognitive performance becomes. However, the comparison between e-sports gamers and amateur gamers leads to a confounding variable. Indeed, e-sports corresponds to a specific game practice based on specific video game genres and on competition only. Thus, there is a need to confirm that play time affects the cognitive performance of amateur players. Recent studies overcame the gamer/non-gamer dichotomy by using ordinal variables [16] or continuous variables [17] to account for gaming time. Kowal et al. (2018) [16] categorized participants into five video game time groups (i.e., 0, 1–7, 8–15, 16–22, and 23+ hours per week) and compared the groups’ inhibition and mental flexibility skills. The findings showed that the group with the highest video game time had the lowest overall reaction times to the Stroop test and the lowest non-switching and switching reaction times to the Trail Making Test. However, it remains unclear whether play time, taken as a continuous variable, underlies cognitive performance. Waris et al. (2019) [17] assessed the relations between time spent playing video games per week and working memory skills. The authors found strong evidence for positive linear relations between weekly video game time and visual working memory and updating skills. However, no significant relation was found considering verbal working memory skills. Therefore, it remains to be confirmed whether play time, taken as a continuous variable, is a significant predictor of overall cognitive abilities.

### Game type–based definition of players

In addition to the dichotomous definition of players, a second limitation that restricts a full understanding of the potential relationship between play and cognitive functions is the lack of consideration of playing practice diversity. Numerous studies have highlighted the great potential of action video games to improve cognitive abilities. Action video games can be defined as video games with time pressure, requiring switching between distributed and focused attention and preventing full task automatization [18]. Bediou et al.’s (2018) [19] meta-analysis showed that playing action video games robustly improves attention and visuospatial cognition. However, any video game genre (e.g., traditional, simulation, strategy, action, or fantasy video games) may also have the potential to improve cognitive performance [3]. For example, one study found that players’ visuospatial skills significantly improved after playing the strategy game Portal 2 for 8 hours [11].

Furthermore, few studies have focused on the effects of non-digital games, such as board games, on cognitive functions. However, a clear-cut distinction exists between video games and board games, as they are defined by unique game features that do not apply equally to both types of games. Inherently, video games correspond to numeric leisure, while board games are mainly analog games. Even though some digitized board games that fully reflect their analog versions exist, they represent only the minority of board games and practices. Moreover, video games are mainly played alone, while board games are played in groups. There is a current trend in which online video game players are playing these games because they allow for interactions between gamers, but these are remote interactions, while board games mostly imply physical and social interactions between the players. In addition, video games are mostly defined by real-time dynamics. Gamers are most often required to make real-time decisions, which are led by the pace of the game. They provide a diversity of actions and environments in a single game, training strategy adaptation skills [20]. Rather, board games are defined by their requirement for social interactions. They allow for the training of specific strategies and actions in a given game, which mainly depend on players’ interactions.

A new research field on board games and cognitive functions recently emerged [21], but the number of studies in this field remains limited. These studies have mostly focused on the effects of traditional board games’ effects on the cognitive and executive abilities of the elderly and children’s cognitive and executive abilities [22, 23]. Traditional board games correspond to abstract strategy games (e.g., chess, checkers, game of Go). Some training programs using this kind of game have been found to enhance working memory, attention, and global executive abilities [4]. Chess practice is positively related to fluid intelligence, short-term memory [24] and decision making performance [25], whereas game of Go practice enhances working memory performance [26]. Playing traditional board games has been shown to be related to a neural reorganization of brain areas associated with attentional control, working memory, and problem solving [27, 28]. Similarly, playing modern board games also seems to improve cognitive and executive abilities.

Compared with traditional board games, modern board games correspond to newer games that offer a wide range of game mechanics (e.g., Ticket to Ride, Splendor, Carcassonne). Playing modern board games has been shown to be related to logical thinking [29], improved fluid intelligence [30], and improved verbal working memory [31]. Moreover, modern board games seem to enhance social abilities, including verbal, relationship, and emotional skills [32]. Although the number of studies on board games and cognitive functions remains limited, board gaming appears to affect cognitive abilities differently compared with video gaming. Board gaming seems to enhance fluid intelligence, verbal working memory, and social performance, whereas video gaming improves attention, visuospatial, working memory, and mental flexibility performance. Therefore, recent literature widely supports the beneficial effects of play on cognitive functions, but the nature of the relationship between game-specific practice and cognitive abilities still needs to be clarified. To our knowledge, no study has examined the cognitive implications of video games and board games in the same model; instead, until now, all studies have examined video gamers’ and board gamers’ cognitive abilities independently. However, the time spent playing video games and board games may be positively related. Players tend to play both game types proportionally, which leads to confounding variables. It remains unclear whether the benefits of play are due to specific game practices (i.e., video gaming and board gaming) or to overall playing practice, regardless of game type. Therefore, further investigations are needed to study the specific cognitive contributions of video games and board games concurrently in the same model.

Considering the recent literature on games and cognitive functions, the remaining question is whether the benefits of play on cognitive functions are due to the overall play time or to specific game types. To address this original question, we conducted an online experiment in which we aimed to overcome the dichotomous categorization of players. To do this, we examine the association between overall and game-specific play times and cognitive functions, considering play times as continuous variables and comparing video game and board game contributions in the same statistical model. More precisely, we compare the relationships between cognitive abilities and overall play time (i.e., the time spent playing video games and board games), the time spent playing video games and the time spent playing board games. Considering the recent literature on play and cognitive functions, we expected a significant relation between overall play time and overall cognitive performance [16], but we expected differentiated relations between game-specific play times and specific cognitive measures [3, 4].

## Materials and methods

The experiment was approved by the General Data Protection Regulation service of University XXX (declaration no. 202186).

### Participants

**Fig 1:**
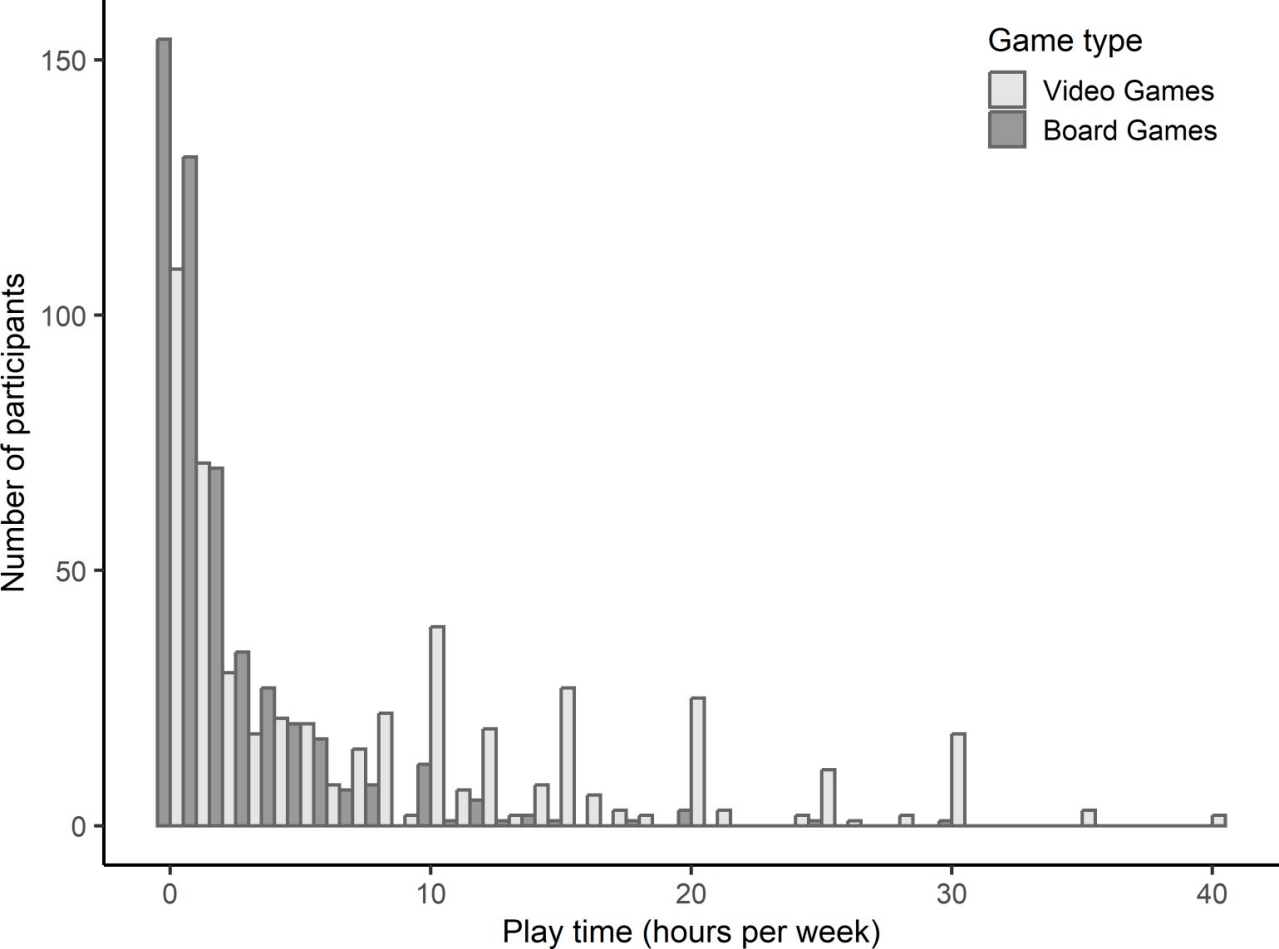
Distribution of hours playing video games and board games per week. Note. Each binwidth = 1 hour.

Four hundred ninety-six French participants (268 women, mean age = 28.08 years) were recruited among undergraduate students, gaming associations, and social networks. All participants gave their written informed consent to participate and received either financial compensation (£8.5) or student course credit to complete their course unit. Individuals who played video games for 0–40 hours per week (M = 7.72, SD = 8.69) and board games 0–30 hours per week (M = 2.37, SD = 3.54), with an overall play time in the range of 0–48 hours per week (M = 10.09, SD = 10.00), were recruited (Fig 1).

Regarding their main leisure activity, 24.2% of the participants reported video games, 4.03% board games, and 71.8% another leisure activity. Considering video gamers only–participants who played video games at least 1 hour per week– 72.2% reported their preferred video games as action video games (e.g., first-person shooter or multiplayer online battle arena). In addition, 27.8% reported non-action video games (e.g., strategy games and management games) as their preferred game. We noted that the most played video games were Leagues of Legends, Animal Crossing, and Mario Kart. For board gamers only–participants who played board games at least 1 hour per week– 71.3% reported their preferred board games as casual board games (e.g., party games and quiz games), and 28.7% reported expert board games (e.g., abstract games and role play games) as their preferred games. We noted that the most frequently played board games were Uno, Monopoly, and chess. Only 12.9% of the participants played digitized board games (–considered board games in this study) at least once a week, and it is important to note that only 11 participants played chess online.

### Cognitive tests

#### Fluid intelligence

Raven’s matrices SPM38 [33], series D and E, were computerized to measure fluid intelligence. The exact same series were used by Bartolucci et al. (2019) [30] to assess the effects of board games on fluid intelligence. Similarly, Raven’s matrices APM were used by James et al. (2011) [34] to assess the effects of video games on fluid intelligence. In this test, participants were presented with a picture composed of eight figures linked by a logical pattern and a missing figure. The task was to find the missing figure that could logically complete the picture among the eight suggested options. The test consisted of 24 experimental trials with increasing difficulty. The number of correct answers was scored.

#### Mental flexibility

The test from Experiment 1 in Monsell et al., 2003 [35] was adapted to assess mental flexibility skills. The exact same task was used by Green et al. (2012) [36] to study action video gamers’ mental flexibility skills. In this test, participants were presented with four types of stimuli (blue squares, red squares, blue circles, or red circles). The task was to classify each stimulus according to its shape (square or circle) or its color (blue or red). The stimuli were displayed successively on a background composed of eight parts, defined by eight equally spaced circle radii. The horizontal radii of the background were thickened to indicate the location of the task switch. Above the horizontal radii, participants classified the stimuli as circle or square (shape condition), whereas under the horizontal radii, participants classified the stimulus as red or blue (color condition). Therefore, the task to be performed was cued by location on the screen, changing every four trials. In the shape condition, participants used the left middle or index finger to press the “Q” key or the “S” key on a computer keyboard to give their answer. In the color condition, they used their right index or middle finger to press the “L” key or the “M” key. As soon as a participant responded by pressing a key, the next stimulus was presented counterclockwise in the next locus.

The test was composed of 32 training trials and 128 experimental trials. The trials in which the instruction changed (i.e., first trials above and below the horizontal radii) corresponded to the switching condition, whereas the trials in which the instruction remained the same corresponded to the non-switching condition. Thus, the switching condition represented 25% of the trials. Accuracy (i.e., proportion of correct answers) and reaction time were recorded in both switching and non-switching conditions. The differences between the accuracy and the reaction times in both switching and non-switching conditions were then computed.

#### Planning

A computerized version of the Tower of London test [37, 38] was used to assess planning skills. Boot et al. (2008) [39] used the same test to examine the relationship between video gaming and planning skills. In this test, participants were presented with a board with three pegs and three colored balls. The balls could be moved by clicking on the pegs and applying the following rules: No more than three balls could be placed on the biggest peg, no more than two balls on the middle peg, and no more than one ball on the smallest peg. The task was to reproduce a specific arrangement of the balls, which was presented at the top right-hand corner of the screen, with the smallest possible number of moves and within 60 seconds. The task included a training trial and 12 experimental trials. The number of correctly reproduced arrangements was scored.

#### Visual working memory

A computerized version of the Corsi block-tapping task was used to measure visual working memory skills [40]. A similar version of the task was used by Hazarika and Dasgupta (2020) [41] to examine the neural correlates of action video gaming in visual working memory tasks. Participants were presented with nine dark blue squares, of which some randomly flashed in bright orange, in a specific order. In the forward condition, participants were required to reproduce the sequence by clicking on the same blocks in the same order, whereas in the backward condition, they were required to reproduce the sequence in reverse order. Both conditions included two training trials and two experimental trials for each sequence length, ranging from two to nine flashing blocks in the forward condition and from two to eight flashing blocks in the backward condition. Participants were informed that the sequence length would increase gradually. When they failed both trials of the same sequence length, the condition ended. The span (i.e., the longest sequence correctly reproduced) was then scored.

#### Verbal working memory

The shortened version of the operation span task was used to assess verbal working memory skills [42]. A similar task was used to assess the relations between action and real-time strategy gaming and verbal working memory skills [43]. In this test, participants were required to remember letter sequences, and as a distractor task, to judge whether arithmetic operations were correct. After each operation-letter sequence, participants were asked to recall the letters in the correct order. The test was composed of two training trials and six experimental trials (two trials for each letter sequence length, ranging from four to six letters). An absolute score (i.e., the number of letter sequences correctly recalled) was recorded.

#### Visuospatial processing

Peltier and Becker’s (2016) [44] visual search task was adapted to assess visuospatial processing skills. A similar task was used in Hubert-Wallander (2011) [45], assessing the visuospatial skills of action video gamers. During this test, participants were required to determine whether the figure included the letter “T” among a set of distractors (letter “L”). Participants were asked to press the “M” key or the “Q” key on a computer keyboard to give their answers. A fixation cross was displayed for 500 ms before each trial. The letters were randomly rotated from their upright positions by 0°, 45°, 90°, 135°, 180°, 225°, 270°, or 315°. The task included two training trials and 24 experimental trials (4 trials– 2 target absent, 2 target present trials–for each set size– 10, 15, 20, 25, 30, or 35 letters). The number of correct answers and the reaction time were recorded.

### Player profiles

#### Play time

Participants’ play times were assessed using two self-reported questions: “How often do you play video/board games?” and “On average, how many hours do you play video/board games per week?” The given responses ranged on a 6-point Likert-type scale from “Never” to “Several times a day” to the first question, and responses were given in hours to the second question. The answer to the first question was used to control the answer to the second question (e.g., participants who answered “Never” to the first question and did not answer “0 hours” to the second were excluded).

#### Game type

Participants’ favorite video and board game genres were recorded using the alternative choice question “Choose your favorite video/board game genre." Responses were chosen from a list of common video game genres (e.g., first-person shooters, multiplayer online battle arenas, and puzzle games) and common board game genres (e.g., party games, abstract games, and quiz games). The most played games were also recorded through the open-ended question "What video game have you played the most in the last 6 months?"

### Procedure

The participants were all tested online from October 2021 to March 2022. They first completed the Raven matrices, Monsell, Tower of London, Corsi block-tapping, operation span, and visual search tasks, and then completed the gaming experience and demographics questionnaire, which lasted approximately 45 minutes. Because the study was conducted online, quality controls were applied to the data. Participants’ time spent on each cognitive task, responses to attention check questions between each test, and feedback reported at the end of the study were assessed. Thus, any participants who completed the study in less than 20 minutes, those who reported being distracted, those who had any cognitive disorders, or those who engaged in drug use were excluded.

### Analysis

Statistical analyses were performed using R [46]. For all cognitive tests, outlier reaction times to correct responses were detected and removed for each participant using three median absolute deviations around the median reaction time [47]. Outliers were also detected based on the visualization of the frequency distributions of correct responses. This was done using histograms [48].

For all statistical tests, we used an alpha level of .05. We conducted regression modeling to examine how participants’ overall play time and game-specific play times (i.e., time spent playing video games vs. time spent playing board games) were related to cognitive performance. Because no theory suggests that the relationship between play time and cognitive functioning is strictly linear, we decided to compute generalized additive models (GAMs) [49]. GAMs capture the non-linear aspects of and the variations in a relation based on flexible, smoothing splines. These splines correspond to the sum of multiple basis functions, each multiplied by a coefficient to fit the data and create the overall shape of the relation. We used the mgcv package in R to compute the GAMs. First, we tested the relation between all cognitive scores and overall play time. Second, we computed multivariate GAMs to test the relations between all the cognitive scores and game-specific play times and to determine the specific predictors of cognitive performance.

The assessed cognitive functions have been shown to be affected by aging (for a review, see Harada et al., 2013 [50]) and education level (for a review, see Lövdén et al., 2020 [51]). Participants’ ages ranged from 18 to 59 years, and their highest degrees ranged from none to a doctoral degree. Thus, we decided to control for participant age and education level. All predictor variables (i.e., time spent playing video games and time spent playing board games) and control variables (i.e., age and education level) were entered into smoothing splines in the multivariate GAMs.

## Results

### Relation between overall play time and cognitive performance

GAMs were computed to assess the relation between all the cognitive measures and overall play time. The estimated degrees of freedom (edf), the p-values, and the coefficients of determination (R^2^) are reported in Table 1.

**Table 1:** Results of the GAMs for overall play time.

Cognitive function	Variable	Overall play time		
		edf	p	adj. R^2^ (%)
**Fluid intelligence**	**Raven matrices test**			
	Number of correct responses	**4.984**	**.001**	**4.1**
**Mental flexibility**	**Monsell task**			
	No-switching–switching accuracy	1.000	.388	0.0
	No-switching–switching reaction time (ms)	**1.000**	**< .001**	**2.5**
**Planning**	**Tower of London task**			
	Number of correct responses	**2.285**	**< .001**	**4.2**
**Visual working memory**	**Corsi block-tapping task**			
	Forward span	**2.705**	**< .001**	**7.1**
	Backward span	**1.000**	**< .001**	**2.7**
**Verbal working memory**	**Operation span task**			
	Absolute score	**3.91**	**< .001**	**4.8**
**Visuospatial processing**	**Visual search task**			
	Number of correct responses	**1.000**	**.030**	**0.8**
	Reactions times (ms)	**1.000**	**.030**	**0.8**

Note. Estimated degree of freedom (edf) greater than 1.0 indicates a non-linear relation.

The overall play time was significantly related to the number of correct responses in the Raven matrices test, the number of correctly reproduced arrangements in the Tower of London test, the forward span in the Corsi block-tapping task, and the number of letter sequences correctly recalled in the operation span task. In addition, the overall play time was significantly and linearly related to the difference between the non-switching and switching reaction times in the Monsell task, the backward span in the Corsi block-tapping task, and the number of correct responses and the reaction time in the visual search task.

### Relation between game-specific play time and cognitive performance

Multivariate GAMs were performed to test whether the time spent playing video games and the time spent playing board games significantly predicted cognitive measures. The time spent playing board games was found to be significantly related to age (edf = 3.865, p < .001) and education level (edf = 4.366, p = .004). Considering these significant relations and the literature on cognitive aging and education, it was necessary to control for participant age and education level. In Model 1, only age and education level were entered into the analysis. In Model 2, all predictor variables (i.e., time spent playing video games, time spent playing board games, age, and education level) were entered into the regression equations simultaneously. These analyses provided an estimate of the additional variance explained by play times when controlling for age and education level. The models’ coefficient of determination (R^2^) and predictors’ estimated degree of freedom (edf) are reported in Table 2.

**Table 2:** Results of the multivariate GAMs for game-specific play times.

Cognitive function	Measure	Model	Variable	edf	p	ajd. R^2^ (%)
**Fluid intelligence**	**Raven matrices test**	**1**	Age	**3.058**	**.015**	**15.8**
	Number of correct responses					
			Education level	**1.492**	**< .001**	
		**2**	Age	**3.162**	**.037**	**17.8**
			Education level	**1.505**	**< .001**	
			Time spent playing video games	**1.882**	**.044**	
			Time spent playing board games	2.220	.172	
**Mental flexibility**	**Monsell task**	**1**	Age	1.673	.413	1.1
	No-switching–switching accuracy		Education level	1.829	.271	
		**2**	Age	1.938	.236	1.6
			Education level	1.839	.286	
			Time spent playing video games	1.682	.413	
			Time spent playing board games	1.434	.595	
	No-switching–switching reaction times (ms)	**1**	Age	**3.518**	**< .001**	**6.4**
			Education level	1.000	.796	
		**2**	Age	**3.624**	**< .001**	**9.1**
			Education level	1.003	.631	
			Time spent playing video games	**1.000**	**< .001**	
			Time spent playing board games	1.611	.379	
**Planning**	**Tower of London task**	**1**	Age	**2.476**	**< .001**	**6.7**
	Number of correct responses		Education level	**1.000**	**.018**	
		**2**	Age	**2.737**	**< .001**	**9.5**
			Education level	**1.000**	**.009**	
			Time spent playing video games	**2.254**	**.002**	
			Time spent playing board games	1.000	.782	
**Visual working memory**	**Corsi block-tapping task**	**1**	Age	**1.413**	**.003**	**4.6**
	Forward span		Education level	**2.012**	**.001**	
		**2**	Age	**1.000**	**< .001**	**10.3**
			Education level	**1.993**	**.001**	
			Time spent playing video games	**2.088**	**< .001**	
			Time spent playing board games	2.093	.164	
	Backward span	**1**	Age	**1.000**	**< .001**	**3.0**
			Education level	**1.523**	**.016**	
		**2**	Age	**1.000**	**< .001**	**6.6**
			Education level	**1.726**	**.035**	
			Time spent playing video games	**1.000**	**< .001**	
			Time spent playing board games	4.018	.184	
**Verbal working memory**	**Operation span task**	**1**	Age	**5.156**	**.002**	**8.8**
	Absolute score					
			Education level	**1.000**	**.012**	
		**2**	Age	**1.000**	**< .001**	**10.5**
			Education level	**1.000**	**< .001**	
			Time spent playing video games	**2.188**	**.018**	
			Time spent playing board games	1.001	.176	
**Visuospatial processing**	**Visual search task**	**1**	Age	3.370	.108	1.2
	Number of correct responses					
			Education level	1.000	.894	
		**2**	Age	3.501	.102	2.0
			Education level	1.000	.790	
			Time spent playing video games	1.000	.064	
			Time spent playing board games	1.428	.504	
	Reaction times (ms)	**1**	Age	**3.347**	**< .001**	**12.2**
			Education level	**1.818**	**.008**	
		**2**	Age	**3.803**	**< .001**	**14.4**
			Education level	**1.237**	**.001**	
			Time spent playing video games	**3.202**	**.018**	
			Time spent playing board games	2.265	.187	

Note. Estimated degree of freedom (edf) greater than 1.0 indicates a non-linear relation.

**Fig 2:**
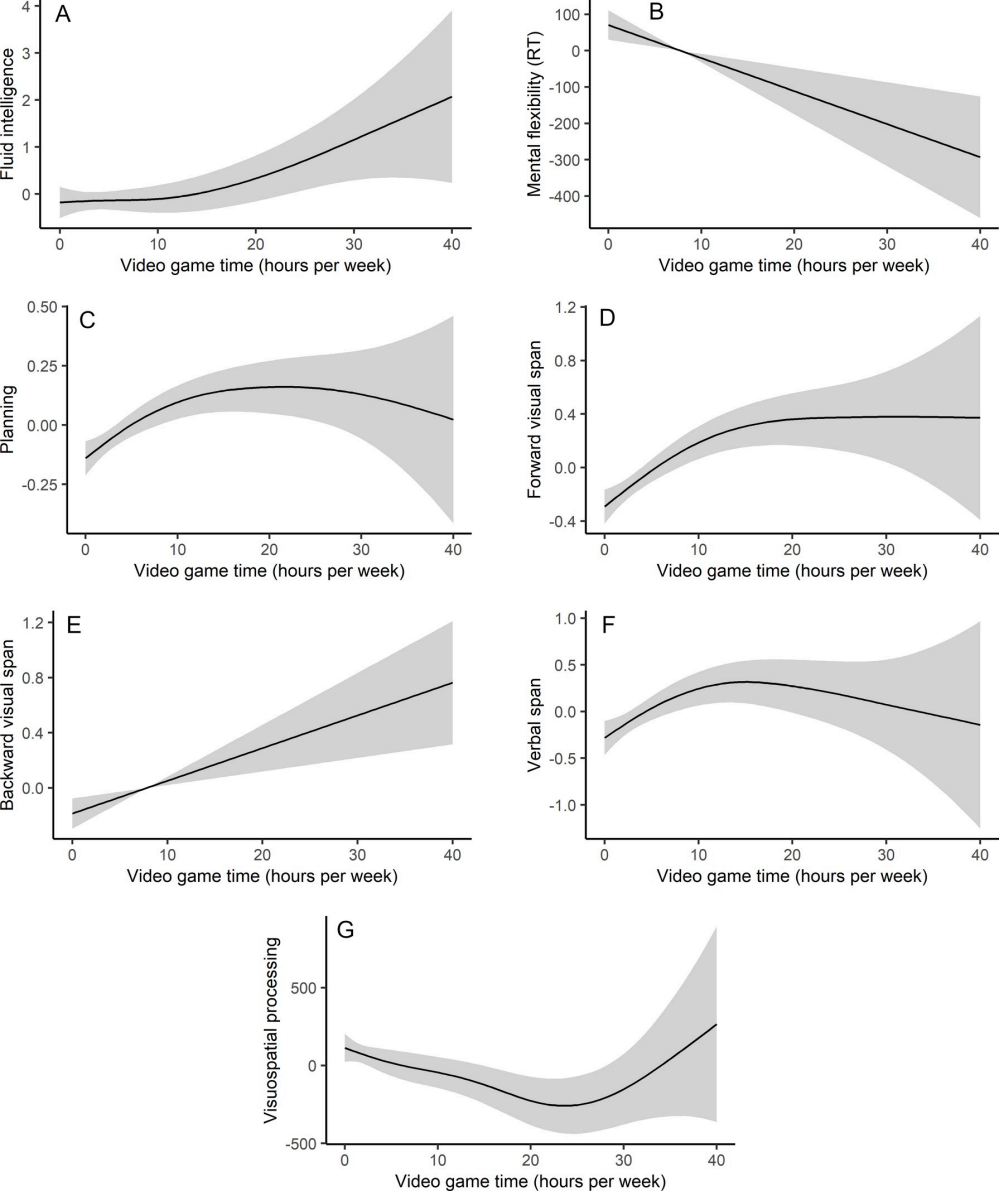
Graphical presentation of GAM for the cognitive measures in relation to the time spent playing video games. Note. Plots with 95% intervals.

As expected, age was found to be a significant predictor of all assessed cognitive functions. Education level was found to be a significant predictor of all cognitive performance, except mental flexibility. Controlling for age and education level, the time spent playing video games significantly predicted the number of correct responses in the Raven matrices test, the difference between switching and non-switching reaction times in the Monsell task, the number of correctly reproduced rearrangements in the Tower of London task, the forward and backward spans in the Corsi block-tapping task, the number of letter sequences correctly recalled in the operation span task, and the reaction times in the visual search task (see Fig 2).

More specifically, mental flexibility and visual working memory skills were found to be linearly related to the time spent playing video games. The more the participants played, the lower their switching cost and the higher their backward visual span (see Fig 2B and 2E). Similarly, fluid intelligence skills were found to increase, along with video game time, especially after 10 hours of gaming per week (see Fig 2A). Planning skills and verbal span were also found to increase with video game time, but this positive relation reached a limit between 10 and 20 hours of video gaming per week (see Fig 2C and 2F). However, visuospatial processing speed was found to increase (i.e., lower reaction times) as video game time increased, reaching a limit around 20 hours of gaming per week and then decreasing in more intensive gamers (see Fig 2G).

Playing practice specifically explained 5.7% of the variance in the visual forward span and 3.6% in the visual backward span, 2.8% of the variance in planning, 2.7% in mental flexibility, 2.2% in visuospatial processing skills, 2% in fluid intelligence, and 1.7% in verbal working memory. However, controlling for age and education level, the time spent playing board games was not related to any cognitive measures.

## Discussion

Given the wide popularity and cognitive benefits of video and board games, the current study aimed to elucidate the relationship between playing practices and cognitive abilities. Until now, players were mainly defined according to a minimum play time and a specific game type, which is not representative of real playing practices. Therefore, we overcame the dichotomous definition of players by examining whether the benefits of play were explained by the overall play time or specific playing activities. To accomplish this, we assessed the relationships between the participants’ overall and game-specific play times and six main cognitive abilities. Our results demonstrated significant relationships between overall play time and cognitive performance and revealed the important implications of video game practice time in cognitive functions.

The GAMs revealed that overall play time was related to all the assessed cognitive performances. In line with our hypothesis, the overall play time predicted fluid intelligence, mental flexibility, planning, visual and verbal working memory, and visuospatial performance. We noted positive non-linear relations between play time and planning skills, verbal span, and forward visual span, as well as positive linear relations between play time and backward visual span and visuospatial processing skills. Regarding reaction times, we noted negative linear relations between play time and mental flexibility and visuospatial processing speed. Therefore, our results showed positive relations between overall play time and both accuracy and efficiency.

Considering mental flexibility, we found only a negative linear relation between the overall play time and the difference between the non-switching and switching reaction times. The more the participants played, the higher their efficiency became, but their accuracy did not show a similar increase. This is in accordance with Dye et al.’s (2009) [52] findings showing that gamers responded faster to several switching tasks without losing accuracy. Overall, our findings are in line with the recent literature on the cognitive benefits of play [3, 4]. Most importantly, we showed a positive relationship between play time and cognitive performance.

Using GAMs allowed for the consideration of the individual diversity of play time (e.g., 0–48 hours per week in our sample). Indeed, the dichotomous categorization of players based on minimum play time often fails to capture the current diversity of playing practices. Moreover, on a theoretical level, a consensus has not yet been reached on the minimum play time required for someone to be defined as a player. In recent studies, players have not had equal play time, and non-players are sometimes casual players, playing up to 8 hours per week [3]. Therefore, our findings showed the importance of considering play time as a continuous variable to determine the benefits of play on cognitive abilities. We confirmed the relationship between play and cognitive functions, and most importantly, we demonstrated the not necessarily linear nature of this relationship.

The implications of game-specific play times for cognitive performance were detailed via multivariate GAMs. The analyses showed that age was a significant predictor of all assessed cognitive functions, and education level was a significant predictor of all cognitive measures except mental flexibility. These results are consistent with numerous studies showing that cognitive functions are affected by age [50] and education level [51]. Comparing this model with age and education level as predictors and a model with age, education level, time spent playing video games, and time spent playing board games as predictors, playing practice was found to significantly explain 1.7% to 5.7% of the variance in different cognitive performance.

Video game practice time was found to uniquely predict all assessed cognitive abilities. In line with recent literature, video game practice was related to mental flexibility, visuospatial processing, and visual working memory skills [6, 18]. The time spent playing video games was negatively and linearly related to the difference between non-switching and switching reaction times in the mental flexibility task. Thus, video game practice time positively predicted the limited cost of task switching on players’ efficiency. However, the time spent playing video games was non-linearly related to visual search task reaction times. Playing video games predicted higher efficiency in visuospatial processing tasks but not necessarily higher accuracy, only for gamers playing up to 20 hours a week. This is in line with the literature showing that gamers respond faster to visuospatial tasks without losing accuracy [53]. However, this relation seems to reverse when playing video games more than 25 hours a week. In addition, the time spent playing video games was positively related to the visual forward and backward spans. Interestingly, the analyses also revealed that the time spent playing video games predicted fluid intelligence, planning and verbal working memory skills.

Few studies exist on the relationship between video gaming and fluid intelligence, planning and verbal working memory [10, 54]. However, recent literature has shown that board gamers demonstrate higher fluid intelligence [30], planning [55] and verbal working memory performance [31]. Indeed, we found that the time spent playing board games was significantly related with fluid intelligence (edf = .2.283, p = .002) and verbal working memory skills (edf = 3.720, p = .020). However, after controlling for age, education level, and video game practice time, the associations between board gaming and fluid intelligence and verbal span were no more significant in GAM analyses. Thus, by comparing the cognitive contributions of video games and board games in the same statistical model, something that has not been done in previous studies, our results highlight the specific relationship between video gaming and fluid intelligence and verbal working memory skills.

The unique implications of video game practice in cognitive functions, even after controlling for age, education level, and board game practice time, could be explained by unique game features. Indeed, compared with board games, video games imply real-time dynamics, such as real-time decision making, which have a great potential to enhance cognitive abilities. Playing video games often leads to a high level of arousal. Gamers are mainly required to maintain and manipulate information from multiple sources and to make rapid decisions. They also need to use their attention skills in a flexible manner by switching between distributed and focused attention. These features could help develop attention and mental flexibility skills [20, 56]. Moreover, video games offer various environments, avoiding full task automatization and fostering new strategies and learning [20]. Gamers can also take advantage of increasing difficulty levels that are adapted to their skills, and they can gather immediate informative feedback, which allows them to adapt their behavior and strategies. Finally, video games are intrinsically rewarding and fun, and these characteristics have been shown to yield cognitive enhancement [57]. Further investigations are now required to define which game features particularly explain the cognitive benefits of video games.

A current issue has to do with the structural similarities existing between games’ mechanisms and cognitive tests. Some authors highlighted that the relationship between gaming and cognitive abilities may only demonstrate the training of specific behavioral responses to stimuli that are shared between games and cognitive tasks (e.g., the requirement for rapid responses to a first-person shooter game and reaction times to a go/no-go task) [3, 58]. However, some studies showed that video gaming can enhance other cognitive skills and activities involving these cognitive functions. Thus, interpreting the relationship between video gaming and cognitive functions based on structural similarities only is challenging. For example, children’s reading speed and accuracy significantly increased after playing Rayman Raving Rabbids action mini games, which could be explained by the significant enhancement of their visuospatial and phonological processing skills [59, 60]. Similarly, playing Unreal Tournament 2004 and Angry Birds significantly enhanced undergraduates’ verbal working memory and mental rotation skills, which could explain the significant improvement in their geometry performance [61]. Therefore, the benefits of video gaming on cognitive abilities cannot be attributed solely to structural similarities between the games and the cognitive tasks.

Board game practice time was not found to predict any of the assessed cognitive abilities. These findings did not confirm our hypothesis of differential relationships between game-specific play times and cognitive abilities. The significant relationship between board game practice time and cognitive measures was no longer significant when age and education were controlled. Indeed, board game practice time was significantly related to age and education level. More educated participants and those aged 30 to 40 years spent more time playing board games. Thus, board game practice seems to represent a specific category of the population, capturing age and education level. Therefore, it is difficult to confirm the specific effect of board games on cognitive functions [4]. The already demonstrated effects in the literature may be due solely to the participants’ age and education. Therefore, an interventional study is needed to determine whether cognitive benefits related to board games exist and how much practice time per week would be needed to achieve this effect. These studies could be conducted with younger participants to control for age and education effects.

Our analyses did not reveal a concomitant implication of video games and board games in terms of cognitive performance. Thus, the common features of games, such as that they are rewarding and fun experiences, do not seem to be sufficient to explain cognitive performance. Once again, these findings highlight the importance of identifying the unique game features involved in the association between play and cognitive functions. Further studies will need to consider game specific features and play times to fully understand the cognitive benefits of play.

### Limitations and perspectives for future research

Although the current study offers novel findings on the association between play time, game type, and cognitive functions, some limitations must be noted. As a first limitation, the participants completed the study online, which allowed a large sample to be recruited. Although the data were checked, and any incomplete data were excluded, participants’ engagement and motivation can be questioned. Recent studies have noted that online participants are not necessarily inattentive during the tasks, but they are more likely to be distracted (e.g., in using their mobile phones, talking to another person in the room, etc.) [62]. Thus, a recommendation for ensuring online participants’ motivation and engagement is to provide informative feedback about their performance [63]; we did this for each task, but it may not have been sufficient.

The second limitation lies in the concerns regarding whether bias existed in the self-reported measures of play time. Some studies have shown that players tend to under-report their time spent playing video games [64] and to over-report their genre-by-genre gaming time [65]. To our knowledge, no similar study has assessed bias in the measures of time spent playing board games. Thus, there is currently no way to know whether video game and board game times are biased in the same way. A solution to ensure that play time is accurately measured is to use online measures. In video games, this could correspond to the play time recorded in video games’ servers [64]. A similar measure could be implemented for board games using diary-based reports or timed play sessions [17].

A third limitation is the use of a dichotomous game-type approach. Although we considered play time as a continuous variable, playing practice was still defined according to a game-type dichotomy (i.e., video games vs. board games). This approach allowed us to compute cognitive predictors while controlling for game-specific play times and gave first results on the comparison of video and board games’ cognitive implications. However, future studies will be needed to build on our findings according to game subtypes (e.g., action games, strategy games, etc.) and obtain evidence on the game-specific features mediating cognitive enhancement. Finally, given the correlational nature of our analyses, we demonstrated the existence of linear and non-linear relations between overall and game-specific play times and cognitive performance. However, our results cannot account for the causality of the cognitive benefits of play. Thus, future training studies will be necessary to determine the causal relations between play and cognitive abilities. As discussed above, these studies will have to implement training based on different play times and different game features to specify the effects of play on cognitive functions.

## Conclusion

Given the global popularity of playing practices and the concerns about intensive gamers’ health, it is important to examine the implications of play time and game type on cognitive functions. Our findings are in line with the recent literature showing that playing is beneficial for cognitive functions; moreover, the findings demonstrate that play time predicts players’ cognitive performance. It seems that video games affect cognitive performance more than board games do, which has never been demonstrated in previous studies. The unique features of this main game type surely explain its specific relationship with cognitive functions. Video games seem to have a greater potential for overall cognitive enhancement because they involve processing various types of information and adapting strategies dynamically and in real time. Future studies on play and cognitive functions will need to account for individual differences among players by considering their play times and the specific features of the games they play.
